# Magnetic resonance imaging of endolymphatic hydrops in Ménière's disease: A comparison of the diagnostic value of multiple scoring methods

**DOI:** 10.3389/fneur.2022.967323

**Published:** 2022-09-26

**Authors:** Heng Xiao, Xiaojing Guo, Huimin Cai, Jianwei Lin, Chenxin Lin, Zheming Fang, Shengnan Ye

**Affiliations:** ^1^Department of Otorhinolaryngology Head and Neck Surgery, Fujian Otorhinolaryngology Institute, The First Affiliated Hospital of Fujian Medical University, Fuzhou, China; ^2^Departments of Imaging, The First Affiliated Hospital of Fujian Medical University, Fuzhou, China

**Keywords:** Ménière's disease, magnetic resonance imaging, endolymphatic hydrops, inner ear structure assignment method, auditory function

## Abstract

**Objectives:**

To compare three methods of scoring endolymphatic hydrops in patients with Ménière's disease in order to assess the correlation between endolymphatic hydrops and auditory characteristics.

**Methods:**

A retrospective study of 97 patients with unilateral definite Ménière's disease (DMD) who underwent contrast-enhanced three-dimensional fluid attenuated inversion recovery (3D FLAIR) MRI. Each patient was scored by the Inner Ear Structural Assignment Method (IESAM), the Saccule to utricle area ratio (SURI), and the Four Stage Vestibular Hydrops Grading (FSVH), according to their corresponding axial images. Cohen's Kappa and intra-class correlation coefficient were used for consistency testing, combined with binary logistic regression analysis, to compare the sensitivity and specificity of the three methods. The degree of hydrops in different stages of MD was compared. The correlation between endolymphatic hydrops in the inner ear sub-units and hearing thresholds was further analyzed.

**Results:**

The intra- and inter-reader reliability for the scoring of endolymphatic hydrops were excellent. The IESAM had a high diagnostic value for identifying definite Ménière's disease (sensitivity: 86.6%, specificity: 97.9%). The hearing thresholds were correlated with the degree of endolymphatic hydrops. Stages 3 and 4 were more significant for the severity of hydrops than stage 1. Within the subgroups of the Ménière's disease patients, compared with the non-hydrops group and the pure vestibular hydrops (V group), the cochlear combined vestibular hydrops group (CV group) had significantly higher auditory thresholds. The amplitude ratio of electrocochleogram was significantly higher in the affected ear than in the healthy ear.

**Conclusion:**

The IESAM is a more sensitive and specific diagnostic scoring method for the diagnosis of DMD. Diagnostic imaging may improve the detection of inner ear hydrops which is correlated with severity of hearing loss. A comprehensive evaluation of the inner ear sub-unit structures maybe necessary.

## Introduction

Ménière's disease (MD) is an inner ear disorder characterized by spontaneous vertigo attacks, fluctuating sensorineural hearing loss (SNHL), tinnitus and/or aural fullness (1). Ménière's disease is most common between the ages of 40 and 60 years, with approximately 50 to 200 per 100,000 adults affected, and a serious impact on the quality of life of affected patients (2). In 2015, the Bárány Association developed simplified diagnostic criteria for MD (3), including two categories: definite and probable MD. At present, the diagnosis of MD is largely based on symptomatology, especially in the early stages of the disease, where symptoms may be atypical and even, in some cases, where the main symptoms overlap with the clinical symptoms of other diseases, such as vestibular migraine (4, 5).

The main pathological feature of MD is endolymphatic hydrops (EH) (6, 7). Currently, gadolinium imaging of the inner ear provides the basis for visualizing endolymphatic hydrops *in vivo* (8). In the last decade or so, the use of magnetic resonance imaging (MRI) for the diagnosis of EH has become increasingly widespread. Endolymphatic hydrops scoring systems and methods are also increasingly available, however, there is still no consensus on the visual scoring of endolymphatic hydrops in Ménière's disease. Therefore, we compared the three most commonly used endolymphatic hydrops scoring methods in the world, evaluating their diagnostic capacity for Ménière's disease as well as the correlation between endolymphatic hydrops and auditory functions.

## Materials and methods

### Patients

From January 2010 to January 2022, a total of 482 patients with symptoms of vertigo, tinnitus, aural fullness, and fluctuating hearing loss underwent gadolinium-enhanced magnetic resonance imaging of the inner ear for the presence of endolymphatic hydrops at our institution.

After obtaining approval from the institutional regulatory board and obtaining written informed consent from the participants, patients were treated according to the routine standard of care. Patients were fully informed prior to tympanic injection, and they could choose to perform bilateral or affected-sided injections at their own discretion. Almost all patients opted for bilateral imaging. The exclusion criteria included (1) bilateral Ménière's disease; (2) a history of previous ear surgery; (3) external or middle ear lesions, large vestibular aqueduct syndrome, or other congenital cochlear malformations; (4) having central vertigo, severe neurological, or psychiatric disorders; and (5) poor contrast imaging; (6) patients in whom MRI was contraindicated. Among them, eight cases of poor imaging were excluded. The clinical diagnosis was made according to the diagnostic criteria for MD established by the Bárány Association in 2015 (3). A total of 97 patients with unilateral definite Ménière's disease (DMD) were included, retrospectively analyzed in conjunction with clinical data. All patients had unilateral disease with the contralateral side being the healthy ear, and bilateral imaging was performed to facilitate binaural comparison. The contralateral normal ear served as a control. The age range was 15–67 years (42 ± 12), female/male = 58:39.

### Imaging acquisition

Gadolinium-diethylenetriaminepentaacetic acid (Gd-DTPA) dimer injection as contrast agent, diluted eight-fold in saline, was administered bilaterally to the tympanic membrane in all 97 patients. After injection, the patient's head was rotated 45° contralaterally and held for 30 min. Twenty four hours later, a three-dimensional fluid-attenuated inversion recovery MRI was performed using a 3 T unit. All MRI examinations were performed using a Verio 3.0T 16 channel head machine (Siemens, Erlangen, Germany), repetition time 6,000 ms, echo time 132 ms, spatial resolution 0.5 × 0.5 × 0.5 mm, isotropic acquisition, scan time = 4 min 16 s. Simultaneous isotropic 3-dimensional-sampling refinement was compared with application optimization using different flip angle evolution (3D-SPC) inversion recovery for fluid decay inversion recovery [repetition time = 6,000 ms, echo time = 388 ms, inversion time (TI) = 2,100 ms, scan time = 5 min 32 s, spatial resolution = 0.7 × 0.7 × 0.7 mm].

### Inner ear analysis

The images were independently assessed by a radiologist and a senior otolaryngologist with more than 5 years of experience in EH imaging review blinded to the clinical findings and the side. The three structures of the inner ear, including the cochlea, vestibule, and semicircular canals, were grouped according to their involvement. All subjects with involvement of only the cochlear region were defined as type C pathological changes, vestibular involvement only and semicircular canals involvement only as type V and Sc, respectively. Similarly, simultaneous cochlear and vestibular involvement was labeled as CV-type, cochlear and semicircular involvement as CSc-type, vestibular and semicircular involvement as VSc-type, and if cochlear, vestibular, and semicircular regions were all involved, patients were labeled as CVSc-type. If no endolymphatic hydrops were detected, the patient was regarded as type N.

### Inner ear structure assignment method

The degree of gadolinium contrast (high signal) filling in the perilymphatic space of the affected ear was observed and assessed. The three structures of the cochlea, vestibule and semicircular canals were assigned different scores according to the following conventions: no filling, partial filling and full filling (non-visible, partially visible, fully visible). A full score of 18 points indicated that there was no endolymphatic hydrops. The lower the scores, the more severe the endolymphatic hydrops, as shown in Table 1 (9). Binary logistic regression combined with subject operating characteristic curve analysis was used to assess the diagnostic capacity of the Inner Ear Structural Assignment Method (IESAM) model for detection of endolymphatic hydrops based on the scores of the three structures of the inner ear.

**Table 1:** Scoring criteria of IESAM in 3D-SPC-FLAIR images^*^.

**Appearence^†^**	**Cochlea**			**Vestibule**	**Semicircular canals**		
	**Base**	**Middle**	**Apex**		**Superior**	**Horizontal**	**Posterior**
Not visible^a^	0	0	0	0	0	0	0
Partially visible^b^	1	1	1	3	1	1	1
Completely visible^c^	2	2	2	6	2	2	3

*Time of inversion (TI) = 2,100 ms.

^†^On 3D-SPC-FLAIR images.

^a^Absence of high-signal contrast medium.

^b^Failure to show high-signal image of entire cochlear canal, or high-signal image of cochlear canal limited to tympanic or vestibular scale, or interrupted high-signal images of semicircular canals, or incomplete high-signal image of vestibule.

^c^All labyrinth structures completely visible.

### Saccule to utricle area ratio

**Figure 1:**
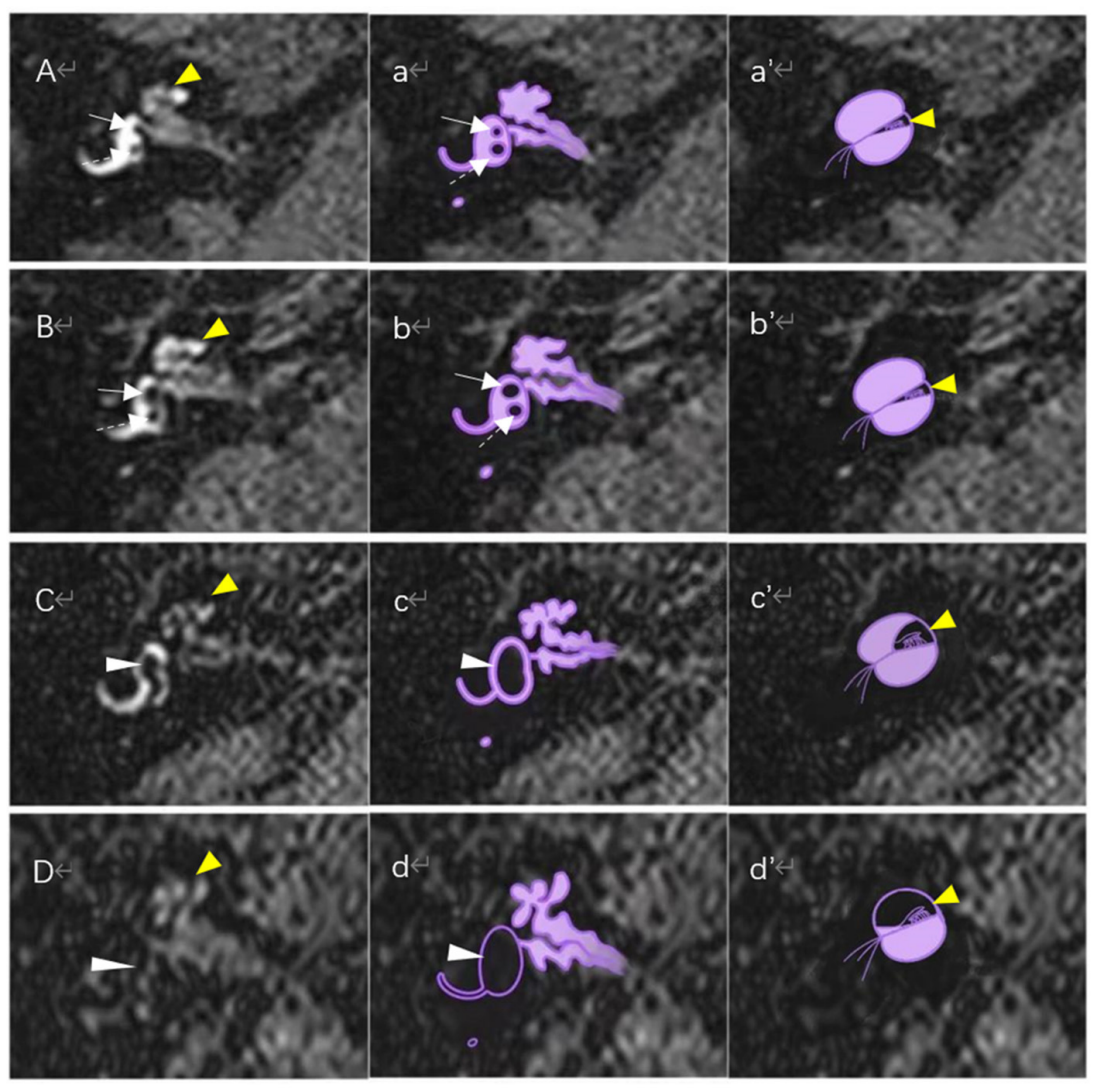
Magnetic resonance imaging of different degrees of endolymphatic hydrops on the axial reference plane of the lower vestibular and schematic diagram of the corresponding vestibular and cochlear endolymphatic hydrops. **(A)** Normal vestibule and normal cochlea: Saccule (white solid-line arrow) and utricle (white dotted arrow) are distinctly separated, and the saccule area is smaller than the area of the utricle; no enlargement of the scala media (yellow arrowhead). **(B)** FSVH grade I (or SURI grade 1) and normal cochlea: saccule (white solid-line arrow) appeared equal or larger than the utricle (white dotted arrow), but is not yet confluent with the utricle; no enlargement of the sacala media (yellow arrowhead). **(C)** FSVH grade II (or SURI grade 2): There is a confluence of the saccule and utricle (white arrowhead) with still a peripheral rim enhancement of the perilymphatic space. The scala media expands toward the vestibular scala vestibuli, which is still visible. **(D)** FSVH grade III (or SURI grade 2): The saccule and utricle are fused (white arrowhead) and peripheral ectolymphatic enhancement is no longer visible; the scala media is enlarged toward the scala vestibuli,which is barely visible. **(a–d)** are schematic diagrams of vestibular hydrops corresponding to **(A–D)**, respectively. **(a′-d′)** are schematic diagrams of cochlear hydrops corresponding to **(A–D)**, respectively.

Using MRI data of the temporal bone region, we graded the EH according to the saccule morphology proposed by Attyé et al. (10). Using the lower part of the vestibule (i.e. 2 mm below the horizontal semicircular canal) as the reference plane. If the saccule area was greater than or equal to the utricle area, i.e., saccule to utricle area ratio (SURI) ≥1, this suggested the presence of endolymphatic hydrops, which was then classified into Grade 1 or Grade 2 according to whether or not the saccule morphology was visible (Figure 1).

### Four stage vestibular hydrops grading

The EH was graded using the four stage vestibular hydrops grading system first proposed by Bernaerts et al. (11). The reference plane for vestibular hydrops is the plane of the lower part of the vestibule (i.e., 2 mm below the horizontal semicircular canal; Figure 1). The four stages of vestibular hydrops are graded as follows:

(i) Normal vestibule: saccule and utricle area are clearly separated; saccule are smaller than utricle and occupy less than half of the vestibular area.
(ii) Grade I: saccule becomes equal to or larger than utricle, but has not yet fused with utricle.
(iii) Grade II: there is a confluence of the saccule and utricle with still a peripheral rim enhancement of the perilymphatic space.
(iv) Grade III: perilymphatic enhancement is not visible and there is a full obliteration of the bony vestibule.

### Auditory function

Pure-tone audiometry was conducted for all patients. The audiometer model used was the OB922 (Madsen, Denmark). Tests were performed in a standard sound isolation shielded room, and the pure tone hearing thresholds of six frequencies in the range of 0.25–8 kHz were measured. PTA thresholds were calculated as the mean value of the four frequency hearing thresholds of 500 Hz, 1 kHz, 2 kHz, and 4 kHz. The mean hearing thresholds of low frequency (250 Hz), middle frequency (500 Hz−2 kHz), and high frequency (4–8 kHz) hearing thresholds were calculated separately. According to the AAO-HNS guideline for PTA-based MD stages (12), participants fell into four groups: 16, 21, 53 and 7 cases in Stage I (PTA ≤ 25 dBHL), II (PTA 26–40 dBHL), III (PTA 41–70 dBHL), and IV (PTA ≥71 dBHL), respectively.

The electrocochleogram (ECochG) test apparatus was an American Nicolay brainstem evoked potentiometer, used in a standard acoustically isolated shielded room with click sound stimulation. The following parameters were set: period 100 μs, scan time 10 ms, filter range 10–3,000 Hz, gain 100 k, polarity alternating wave, rate 11.1 times/s, intensity 95 dBnHL. The recording electrode was placed in the center of the tight tympanic membrane, the ground electrode was placed in the brow, and the reference electrode was placed in the contralateral mastoid process. Diagnostic basis: -SP/AP ratio ≥0.4 was considered abnormal (13).

### Statistical analysis

Data were analyzed using IBM SPSS Statistics 26.0 software and R software (3.6.3 version). Data for categorical and parametric variables were expressed as percentages and mean ± standard deviation (SD) or median (P25, P75), respectively. Data from normal and skewed distributions were compared using analysis of variance (ANOVA) and Mann–Whitney *U*-test, respectively. The intra- and inter-reader reliability of endolymphatic hydrops levels were estimated using Cohen's kappa and Intra-class correlation coefficients (ICC). The sensitivity and specificity of each endolymphatic hydrops scoring methods were calculated. The area under the ROC curve for three scoring methods and the differences in the degree of endolymphatic hydrops between MD stages were performed using the Bonferroni correction to adjust for the multiple comparisons. The correlation between endolymphatic hydrops and auditory function was determined using Spearman's correlation. Tukey HSD *post hoc* test was used to adjust for multiple comparisons of hearing thresholds across groups. *p* < 0.05 was considered statistically significant.

## Results

Clinical features of all patients are illustrated in Table 2. The intra- and inter-reader reliability of endolymphatic hydrops scores were good (0.94 < kappa < 0.96, ICC = 0.98; Table 3).

**Table 2:** Clinical features of all patients.

	**MD (*n* = 97)**	**HC (*n* = 97)**
Age, mean (SD)	42.0 (11.9)	42.0 (11.9)
**Sex**, ***n*** **(%)**
Male	39 (40.2%)	39 (40.2%)
Female	58 (59.8%)	58 (59.8%)
**Affected ear**, ***n*** **(%)**
Left	62 (63.9%)	62 (63.9%)
Right	35 (36.1%)	35 (36.1%)
**Hearing threshold, mean (SD)**
LF	48.9 (17.1)	17.8 (5.6)
MF	45.6 (19.0)	16.8 (4.4)
HF	50.1 (21.7)	19.5 (11.3)
PTA	45.7 (18.6)	17.4 (4.8)
Type of hydrops pathology*, ***n*** **(%)**
C	3 (3.1%)	0 (0%)
V	22 (22.7%)	2 (2.1%)
Sc	0 (0%)	2 (2.1%)
CV	34 (35.1%)	0 (0%)
CSc	0 (0%)	2 (2.1%)
VSc	3 (3.1%)	2 (2.1%)
CVSc	24 (24.7%)	0 (0%)
*N*	11 (11.3%)	89 (91.8%)

*Classification based on the site of endolymphatic hydrops involvement on imaging.

LF, low frequency; MF, medium frequency; HF, high frequency; PTA, pure-tone average; C only cochlear involvement; V only vestibular involvement; Sc only semicircular canals involvement; CV, simultaneous cochlear and vestibular involvement; CSc, simultaneous cochlear and semicircular canals involvement; VSc, simultaneous vestibular and semicircular canals involvement; CVSc, cochlear, vestibular, and semicircular regions were all involved; N, no endolymphatic hydrops.

**Table 3:** Sensitivity and specificity of three scoring methods for the diagnosis of Ménière's disease by magnetic resonance endolymphatic hydrops imaging.

**Scoring methods**	**Sensitivity**	**specificity**	**AUC**	**95% CI**	**Inter-reader reliability**	**Intra-reader reliability**
IESAM^a^	86.6	97.9	0.94	0.90–0.98	0.98	0.98
SURI^b^	84.5	94.8	0.90	0.86–0.95	0.96	0.95
FSVH^b^	84.5	93.8	0.90	0.8–50.95	0.95	0.94

^a^Consistency testing of quantitative data using ICC.

^b^Consistency testing of categorical data using Cohen's kappa.

FSVH, four stages of vestibular hydrops grading; IESAM, inner ear structure assignment method; SURI, Saccule to utricle area ratio.

**Figure 2:**
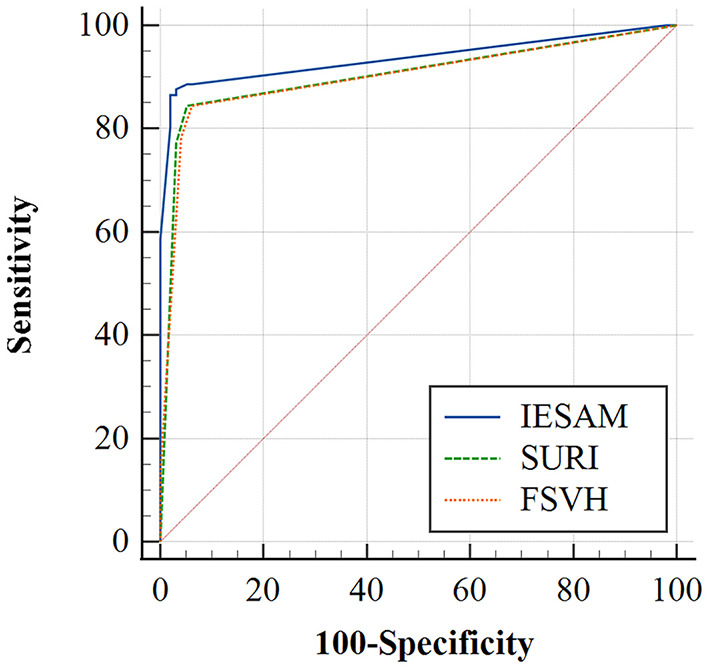
Receiver operating characteristic curves for three scoring methods of endolymphatic hydrops with definite Ménière's disease.

Of the three endolymphatic hydrops scoring methods, the IESAM displayed the highest diagnostic efficacy. Across all MD patients, the sensitivity and specificity of IESAM were 86.6 and 97.9%, respectively (Figure 2). Table 3 lists the sensitivity and specificity of each assessment method to correctly diagnose MD. The difference in the area under the ROC curve between IESAM and SURI or Four Stage Vestibular Hydrops Grading (FSVH) diagnostic methods was statistically significant in MD patients (adjusted *p* value < 0.05), and the difference in the area under the ROC curve between SURI and FSVH was not statistically significant (adjusted *p* value > 0.05; Table 4).

**Table 4:** Multiple comparison of the area under the ROC curve of the three imaging scoring methods.

**Group i**	**Group j**	**Difference between areas**	***z* statistic**	***p*-Value**	**95% confidence interval**
IESAM	SURI	0.0329	2.585	0.0097*	0.00797 to 0.0579
IESAM	FSVH	0.0354	2.722	0.0065*	0.00991 to 0.0609
SURI	FSVH	0.00244	0.486	0.6272	−0.00742 to 0.0123

^*^Bonferroni correction adjusted p-value and “significant” results. Bold value represents statistically significant p-value adjusted < 0.05.

**Figure 3:**
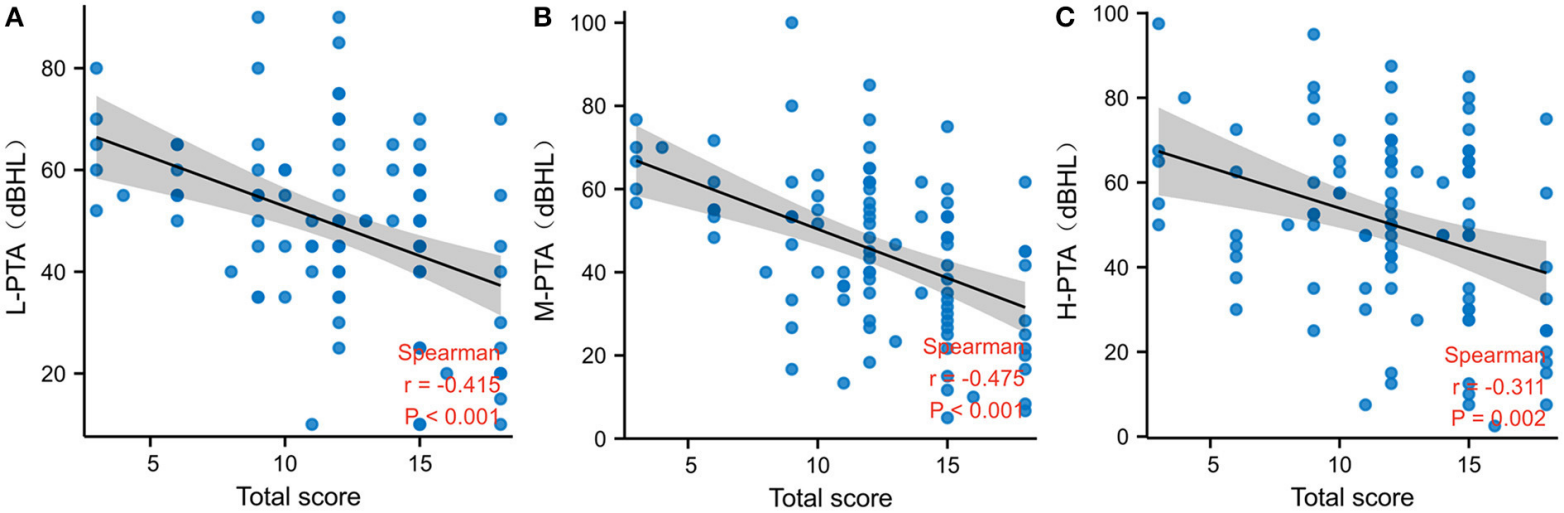
Correlation between IESAM total scores and pure tone hearing thresholds in the Ménière's patients. **(A)** Negative correlation between low-frequency hearing thresholds and total scores; **(B)** Negative correlation between mid-frequency hearing thresholds and total scores; **(C)** Negative correlation between high-frequency hearing thresholds and total scores. L-PTA low frequency pure tone hearing thresholds; M-PTA medium frequency pure tone hearing thresholds; H-PTA high frequency pure tone hearing thresholds.

**Figure 4:**
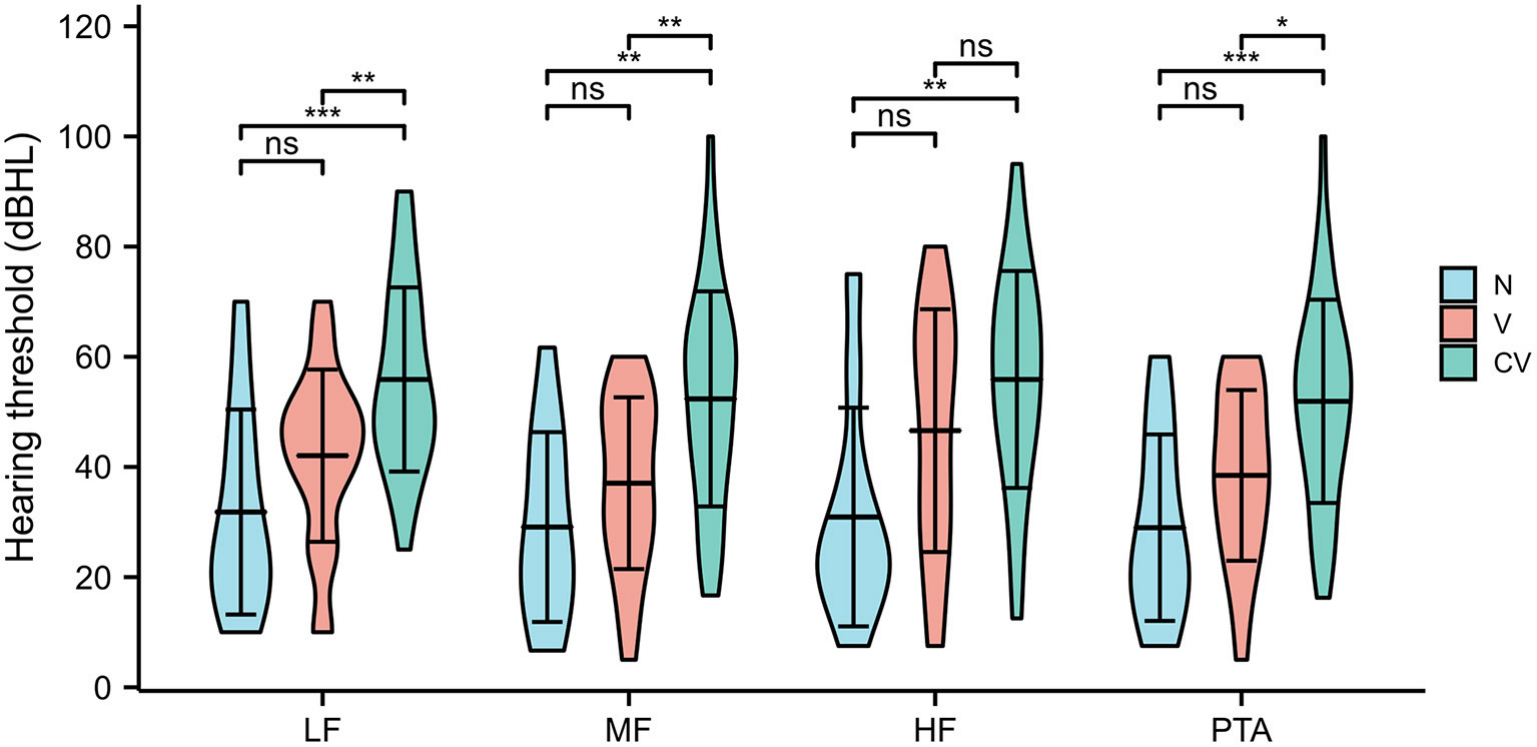
Relationship between different types of hydrops and hearing thresholds at each frequency in Ménière's patients. LF, Low Frequency, MF, Medium Frequency, HF, High Frequency, *N*, no endolymphatic hydrops, V only vestibular region with endolymphatic hydrops, CV cochlear and vestibular hydrops. Ns, *p* ≥ 0.05; *p ≤ 0.05; **p < 0.01; ***p < 0.001. *p*-values were adjusted by the Tukey HSD method for multiple comparisons.

Of the 97 patients with Ménière's disease, IESAM was used to analyze the correlation between endolymphatic hydrops scores and the auditory function. The different frequency hearing thresholds (low, medium and high) were negatively correlated with the total scores of endolymphatic hydrops (Figure 3, *p* < 0.05). After staging of 97 patients according to guidelines, there was a statistically significant difference in the distribution of endolymphatic hydrops among the four stages (*H* = 18.77, *p* < 0.001). The Bonferroni method was used to correct the significance level, and the results showed that the severity of hydrops in stage 3 and stage 4 was greater than that in stage 1, and the difference was statistically significant (adjusted *p* value = 0.001, adjusted *p* value = 0.006, respectively; Supplementary Table 1). There were no statistically significant differences in the degree of intramuscular hydrops between the remaining groups (adjusted *p*-value > 0.05). As shown in Figure 4, the results showed that across the four subgroups of low-frequency, mid-frequency, high-frequency, and PTA, the hearing thresholds of CV type was significantly higher than that of N type patients, and the differences were statistically significant (Tukey HSD test, *p* < 0.001, *p* = 0.001, *p* = 0.003, *p* < 0.001, respectively). But there was no statistical difference between N and V type pure tone hearing thresholds (adjusted *p* > 0.05). Across the three subgroups of low-frequency, mid-frequency, and PTA, the differences in CV-type and V-type hearing thresholds were statistically significant (Figure 4, Tukey HSD test, *p* = 0.004, *p* = 0.004, and *p* = 0.011, respectively), and in the H-PTA subgroup, the differences in CV-type and V-type hearing thresholds were not statistically significant (*p* = 0.317).

Among the 97 patients with MD, there were 43 patients with EcochG examination. The amplitude ratio of EcochG was significantly higher in the affected ear than in the healthy ear (Mann–Whitney *U*-test, *p* < 0.001). The Spearman correlation analysis indicated that there was no correlation between the degree of endolymphatic hydrops in the affected ear and EcochG amplitude ratio (*r* = −0.229, *p* = 0.166). There was no correlation between cochlear hydrops, vestibular hydrops and ECochG amplitude ratio (*r* = −0.314, *p* = 0.055, *r* = −0.099, *p* = 0.555, respectively).

## Discussion

There are many scoring methods of endolymphatic hydrops with Ménière's disease, visual assessment methods provide clinicians with convenient tools to determine the presence of endolymphatic hydrops, among these methods is the IESAM scoring method first proposed by Fang et al. (9), the SURI method described by Attyé et al. (10) and the FSVHS method proposed by Bernaerts et al. (11). We compared the sensitivity and specificity of the three scoring methods in the diagnosis of DMD. Kappa values >0.80 and ICC values >0.75 were generally considered to have good agreement (14), this study demonstrated high inter-reader and intra-reader reliability. Among these scoring methods, IESAM demonstrated optimal imaging diagnostic capacity for the detection of DMD. Our study showed that delayed gadolinium contrast-enhanced 3DFLAIR magnetic resonance imaging is a reliable and accurate technique for the diagnosis of endolymphatic hydrops using a variety of scoring methods. This is the first study to summarize and compare the top, commonly used assessments for diagnostic imaging of endolymphatic hydrops.

Our present study indicated that the IESAM was the better diagnostic imaging method for DMD among the three, with a sensitivity and specificity of 86.6 and 97.9%, respectively. This is consistent with the previous findings of Fang et al. (9). We subsequently hypothesized that the different stages of endolymphatic hydrop development in patients with Ménière's disease affect all three structures of the cochlea, vestibule, and semicircular canals of the inner ear. A comprehensive assessment of the implicated sites of endolymphatic hydrops was performed, with particular attention to the three sub-unit structures of the cochlea, vestibule, and semicircular canals. The pathogenesis of endolymphatic hydrops in Ménière is currently unclear, and a comprehensive assessment of the structure of the inner ear sub-units will facilitate future step-by-step studies of the mechanisms of Ménière's disease in conjunction with imaging and clinical features. We therefore posit that IESAM scoring method may aid in the clinical diagnosis of MD, though more robust testing is additionally required in future studies.

The total scores of endolymphatic hydrops was negatively correlated with the low-, mid- and high-frequency hearing thresholds, suggesting that the severity of hearing loss may indirectly reflect the severity of endolymphatic hydrops. We hypothesize that because endolymphatic hydrops often start in the cochlea and progresses to the saccule, the lesion worsens when the saccule compliance decreases, shedding the saccule otolith and causing obstruction of ductus reuniens (15). As the degree of endolymphatic hydrops increases, it affects the function of inner and outer hair cells as well as the ionic concentration of endolymphatic fluid. The subsequent failure of endolymphatic regulation in the inner ear leads to cochlear damage, such as loss of short cilia in outer and inner hair cells, early changes in spiral ligament fibroblasts or changes in cell morphology- causing hearing loss. This supports the correlation between the severity of endolymphatic hydrops and hearing. This is consistent with the findings of Kahn et al. (16).

The result indicated that the different frequency hearing thresholds (low, medium and high) were correlated with the total scores of endolymphatic hydrops, while the *p*-value was insignificant. According to the stages of diagnostic criteria for MD by AAO-HNS, We found that the PTA of stage 3 and stage 4 was significantly higher than that of stage 1. With the development of hydrops, its influence on the inner ear is more significant, however, MD with mild hearing loss did not show any difference in the degree of hydrops. We speculate that this may be related to the compliance of inner ear subunits. The degree of hydrops in the inner ear increases with the MD stage, and the corresponding pressure in the inner ear membrane labyrinth increases. When the buffering effect of the inner membrane labyrinth is destroyed, it will cause hydrops in the corresponding subunit structure, resulting in more severe hearing loss or vertigo symptoms (17, 18). We further evaluated pure-tone hearing thresholds differences between various groups of Ménière's patients with differentially affected inner ear sub-units. The results of the this study suggest that the CV sub group displayed significantly higher hearing thresholds at all frequencies compared to the MD patients with normative inner-ear structures (N) group. This is consistent with what has been observed clinically. The CV group displayed significantly higher hearing thresholds at low and mid frequencies compared to the V group, suggesting that hearing impairment is more pronounced in Ménière's disease when the cochlea is involved in endolymphatic hydrops. In the cochlea, the most severe endolymphatic fluid accumulation occurs first in the apical turn of the cochlea, followed by the middle and basal turns. As the basilar membrane in the cochlea vibrates in response to an acoustic stimulus, the outer hair cells amplify the stimulus and transmit fluid vibrations to the sound-sensitive inner hair cells. The location of the maximum basilar membrane vibration depends on the frequency of the detected sound. Low-frequency waves are predominantly distributed in the apical turn of the cochlea, while high-frequency waves mainly affect the basal turn of the cochlea. The basilar membrane at the apex of the cochlea is wider and softer than the basilar membrane at the bottom of the cochlea (1). The result is that the expansion of the basement membrane in the EH begins with the apical turn and its corresponding hearing loss. Therefore, cochlear hydrops is often associated with hearing loss. These results were consistent with the previous findings of Zhang et al. (19, 20).

The more commonly used diagnostic method to determine endolymphatic hydrops in Ménière's disease patients is the electrophysiologically-based EcochG, whose diagnostic criteria are: -SP/AP ≥0.4. Pressure changes in the endolymphatic fluid cause the basement membrane to move toward the Scala tympani, generating -SP due to asymmetric basement membrane vibrations. Varying degrees of membranous labyrinth hydrops increase the amplitude of the alternating short sound-induced negative summation potential (SP), leading to an increase in the -SP/AP amplitude ratio (≥0.4). In the present study, the amplitude ratio of EcochG was significantly higher in the affected ear than in the healthy ear, suggesting an abnormality in the affected cochlea, which is consistent with the previous findings (21). Our study showed that there was no correlation between the degree of endolymphatic hydrops and EcochG amplitude ratio in the affected ear. We believe that the amplitude ratio may correlate with the severity and site of endolymphatic hydrops. While this study indicated that the degree of cochlear hydrops did not correlate with the amplitude ratio. So we consider that this may be related to the relatively low sample of vestibular function studies performed in this cohort, a limitation of this study which will be further improved in subsequent studies.

In our study, we excluded 8 patients with poor contrast imaging. We assume that these eight patients may have poor permeability of round window membrane (22, 23), or it is quite possible that the perilymphatic space in severe EH patients is extremely compressed, leading to a significant reduction in the diffusion route of Gd-DTPA. As a result, the perilymph could not be visualized, so we excluded these. In addition, one of the limitations of the current study is the retrospective design and the tertiary care hospital, where patients were only examined according to their need for disease diagnosis. Thus, not all patients underwent ECochG test, with the exception of pure tone audiometry. Another limitation was the small number of cases of ECochG test in this study. In addition, long-term follow-up data for longitudinal evaluation was outside the scope of the current study, which we expect to improve in a follow-up study.

## Conclusion

The IESAM is a imaging assessment method with optimal sensitivity and specificity for the diagnosis of DMD. Diagnostic imaging may improve the detection of endolymphatic hydrops and subsequently indirectly reflect the severity of hearing loss. A comprehensive evaluation of the inner ear sub-unit structure maybe necessary.

## Data availability statement

All datasets generated for this study are included in the article/Supplementary material, further inquiries can be directed to the corresponding author.

## Ethics statement

The studies involving human participants were reviewed and approved by the Ethics Committee of the First Affiliated Hospital of Fujian Medical University. The patients/participants provided their written informed consent to participate in this study.

## Author contributions

HX, XG, and SY designed and coordinated the study. HX and XG analyzed the data and wrote the manuscript. HC, JL, and CL supervised the data collection. HX, XG, and ZF performed the data collection. HX and SY interpreted findings and reviewed the manuscript. All authors contributed to the article and approved the submitted version.

## Conflict of interest

The authors declare that the research was conducted in the absence of any commercial or financial relationships that could be construed as a potential conflict of interest.

## Publisher's note

All claims expressed in this article are solely those of the authors and do not necessarily represent those of their affiliated organizations, or those of the publisher, the editors and the reviewers. Any product that may be evaluated in this article, or claim that may be made by its manufacturer, is not guaranteed or endorsed by the publisher.
