# Supplementary material for: Magnetic resonance imaging of endolymphatic hydrops in Ménière's disease: A comparison of the diagnostic value of multiple scoring methods
Source: Front Neurol. 2022 Sep 26;13:967323. doi: 10.3389/fneur.2022.967323 (PMC9559191; doi:10.3389/fneur.2022.967323)
Supplement: Supplementary file 1 [file Table_1.DOCX]

Supplementary Material

# Supplementary Table

**Supplementary Table 1** Comparison of hydrops scores of patients with definite Ménière's disease at different stages

| Stage i- Stage j | Test statistics | standard error | p value | adjusted p-value |
| --- | --- | --- | --- | --- |
| 1-2 | 18.369 | 9.199 | 0.046 | 0.275 |
| 1-3 | 30.693 | 7.907 | 0.000 | **0.001** |
| 1-4 | 41.607 | 12.562 | 0.001 | **0.006** |
| 2-3 | 12.324 | 7.148 | 0.085 | 0.508 |
| 2-4 | 23.238 | 12.098 | 0.055 | 0.329 |
| 3-4 | 10.914 | - | 0.328 | 1.000 |

^a^ p-value adjusted by the Bonferroni, adjusted p-value <0.05 was considered statistically significant.
